# EZH2 and POU2F3 Can Aid in the Distinction of Thymic Carcinoma from Thymoma

**DOI:** 10.3390/cancers15082274

**Published:** 2023-04-13

**Authors:** Julia R. Naso, Julie A. Vrana, Justin W. Koepplin, Julian R. Molina, Anja C. Roden

**Affiliations:** 1 Department of Laboratory Medicine and Pathology, Mayo Clinic, Rochester, MN 55902, USA; naso.julia@mayo.edu (J.R.N.); vrana.julie@mayo.edu (J.A.V.); koepplin.justin@mayo.edu (J.W.K.); 2 Division of Medical Oncology, Mayo Clinic, Rochester, MN 55902, USA; molina.julian@mayo.edu

**Keywords:** thymic carcinoma, thymoma, immunohistochemistry, EZH2, POU2F3

## Abstract

**Simple Summary:**

The morphologic distinction of thymic carcinomas from thymomas can be challenging but is important as thymic carcinomas tend to be associated with worse patient outcomes. We assessed whether immunohistochemistry for two proteins, EZH2 and POU2F3, could distinguish thymic carcinoma and thymoma. We compared our results with markers previously proposed for this purpose, including CD117 and CD5. POU2F3 (≥10% hotspot staining), CD117, and CD5 staining was seen in 51%, 86%, and 35% of carcinomas, respectively, and in none of the type A thymomas or micronodular thymomas with lymphoid stroma that were tested. EZH2 staining in ≥80% of tumor cells was present in most carcinomas and in only one subtype (type B3) of thymoma. All thymic carcinomas had EZH2 staining in >10% of tumor cells, such that EZH2 staining in ≤10% of tumor cells could help exclude carcinoma. EZH2 and POU2F3 immunohistochemistry may therefore be useful for distinguishing thymic carcinoma from thymoma.

**Abstract:**

Thymic carcinoma is an aggressive malignancy that can be challenging to distinguish from thymoma using histomorphology. We assessed two emerging markers for these entities, EZH2 and POU2F3, and compared them with conventional immunostains. Whole slide sections of 37 thymic carcinomas, 23 type A thymomas, 13 type B3 thymomas, and 8 micronodular thymomas with lymphoid stroma (MNTLS) were immunostained for EZH2, POU2F3, CD117, CD5, TdT, BAP1, and MTAP. POU2F3 (≥10% hotspot staining), CD117, and CD5 showed 100% specificity for thymic carcinoma versus thymoma with 51%, 86%, and 35% sensitivity, respectively, for thymic carcinoma. All POU2F3 positive cases were also positive for CD117. All thymic carcinomas showed >10% EZH2 staining. EZH2 (≥80% staining) had a sensitivity of 81% for thymic carcinoma and a specificity of 100% for thymic carcinoma versus type A thymoma and MNTLS but had poor specificity (46%) for thymic carcinoma versus B3 thymoma. Adding EZH2 to a panel of CD117, TdT, BAP1, and MTAP increased cases with informative results from 67/81 (83%) to 77/81 (95%). Overall, absent EZH2 staining may be useful for excluding thymic carcinoma, diffuse EZH2 staining may help to exclude type A thymoma and MNTLS, and ≥10% POU2F3 staining has excellent specificity for thymic carcinoma versus thymoma.

## 1. Introduction

Thymic carcinomas are rare malignancies that behave more aggressively than thymomas [1,2]. The distinction of thymic carcinoma from thymoma is therefore critical for accurate prognostication and clinical management. However, there is considerable interobserver variability in the morphologic classification of these tumors, with type A thymoma, type B3 thymoma, and micronodular thymoma with lymphoid stroma (MNTLS) most commonly being considered in the differential diagnosis for thymic carcinoma [3]. Type A thymoma and type B3 thymoma are lobulated neoplasms that are epithelial-predominant with no or only a few scattered thymocytes. The neoplastic cells are bland and oval or slightly spindled in type A thymoma, and polygonal with a spectrum of cytologic atypia in type B3 thymoma. MNTLS is characterized by nodules and strands of neoplastic, bland-appearing, oval, epithelial cells in a background of B-lymphocytes focally forming lymphoid follicles with only scattered thymocytes. In contrast, thymic carcinoma lacks the lobulated architecture and usually shows irregular nests of neoplastic cells in a desmoplastic stromal reaction. While the neoplastic cells often exhibit higher grade atypia, sometimes they can be deceptively bland.

The commonly used immunohistochemical markers CD5 and CD117 have only moderate sensitivity (33–73% and 65–85%, respectively) and imperfect specificity (72–100% and 85–100%, respectively) for the distinction of thymic carcinoma from thymoma [4,5,6,7,8]. In general, either high sensitivity or high specificity can be useful for a diagnostic marker. For instance, if positive staining with a marker has high specificity for carcinoma but only moderate sensitivity, positive staining can be interpreted with great confidence as supporting a diagnosis of carcinoma, whereas negative staining may be discounted as uninformative, as carcinomas could also show negative staining. Using a panel of highly specific markers may increase the proportion of cases with positive staining for at least one marker, allowing a greater proportion of cases to be classified. We recently showed that a panel of CD117, BAP1, mTAP, and TdT was able to predict 89% of thymic carcinomas and 78% of type A and B3 thymomas and MNTLS [9], leaving room for improvement in immunohistochemical panels for this differential diagnosis. Moreover, as thymic carcinomas are aggressive malignancies often requiring multimodality therapy, the identification of molecular markers present in even a subset of thymic epithelial tumors (TETs) may be valuable in guiding selection of targeted therapies for those malignancies.

Two promising candidates for the immunohistochemical distinction of TETs are the histone methyltransferase Enhancer of Zeste Homolog 2 (EZH2) and the transcription factor POU class 2 homeobox 3 (POU2F3). EZH2 is involved in thymic development [10]. To our knowledge, only a single study has investigated EZH2 immunoreactivity for distinguishing thymic carcinoma from thymoma [4]. This prior study used exclusively tissue microarrays. The authors found that EZH2 had 89% sensitivity and 100% specificity for thymic squamous cell carcinoma (*n* = 27) versus type B3 thymoma (*n* = 26). EZH2 has not been studied in whole tissue sections of type B3 thymomas or thymic squamous cell carcinomas and has also not been investigated in type A thymoma, MNTLS, or non-squamous and non-neuroendocrine subtypes of thymic carcinoma, such as undifferentiated, mucoepidermoid, lymphoepithelial, sarcomatoid, and adenosquamous carcinomas of the thymus. Accurate assessment of the proportion and subtypes of TETs with EZH2 immunoreactivity may also be valuable in predicting which tumors may respond to EZH2 inhibitors.

POU2F3 is a master regulator of tuft cell identity and may play a role in multiple malignancies [11,12]. Tuft cells have been identified in the thymic medulla [13,14], and evidence of POU2F3 expression in thymic squamous cell carcinomas supports the notion that these malignancies may acquire a tuft cell phenotype [15,16]. To our knowledge, there are only two reports on POU2F3 staining in thymic carcinoma or thymoma, both by the same group [15,16]. These studies used tissue microarrays and a limited number of whole slide sections. The thymic carcinomas in these studies were predominantly of squamous cell carcinoma subtype with only one thymic adenocarcinoma and one thymic mucoepidermoid carcinoma.

Independent validation on larger sample sizes, rare thymic carcinoma subtypes, and whole tissue sections remains to be performed for both EZH2 and POU2F3 in TETs. The performance of immunostains may differ between laboratories, making independent validation of markers a critical step in their integration into clinical practice. Whole slide sections are necessary to appreciate intratumoral heterogeneity in staining, and to understand what may be expected in clinical practice. Accurately interpreting stains performed on rare morphologic subtypes also requires an understanding how markers are expected to perform on those subtypes, which given their unique biology may show distinct staining patterns.

We here provide, to our knowledge, the first independent assessment of these markers since their initial proposal for the distinction of thymic carcinoma from thymoma, using exclusively whole slide sections and including adenocarcinoma, undifferentiated carcinoma, mucoepidermoid carcinoma, lymphoepithelial carcinoma, sarcomatoid carcinoma, and adenosquamous carcinoma of the thymus in addition to type A and B3 thymoma, MNTLS, and thymic squamous cell carcinoma. We present evidence that POU2F3 has moderate sensitivity and excellent specificity for distinguishing thymic carcinoma from thymoma. We found that diffuse EZH2 immunoreactivity was highly specific for thymic carcinoma versus type A thymoma and MNTLS but not type B3 thymoma, whereas the absence of EZH2 immunoreactivity could help to exclude carcinoma.

## 2. Materials and Methods

### 2.1. Patients

Thirty-seven thymic carcinomas and forty-four thymomas were identified from an institutional database of TETs at Mayo Clinic Rochester; all thymomas and thirty-two of the carcinomas were described previously [9]. All cases were reviewed and diagnoses were confirmed by a thoracic pathologist (ACR), following the diagnostic criteria of the 2021 WHO classification [17]. TET were staged according to the 8th AJCC/UICC staging system [18]. Medical records were searched for demographics and clinical features of patients. This study was approved by the Mayo Clinic institutional review board (IRB 10-003525).

### 2.2. Immunohistochemistry

All immunohistochemistry (IHC) was performed using 4 µm formalin-fixed paraffin-embedded whole slide tissue sections. Antibodies used for IHC were as follows: EZH2 (clone D2C9, Cell Signaling Technology, Danvers, MA, USA), POU2F3 (polyclonal HPA019652, Atlas Antibodies, Bromma, Sweden), CD117 (YR145, Cell Marque, Rocklin, CA, USA), CD5 (SP19, Cell Marque), TdT (clone SEN28, Leica Biosystems, Newcastle, UK), BAP1 (clone C-4, Santa Cruz Biotechnology, Dallas, TX, USA), and MTAP (2G4, Abnova, Taipei, Taiwan). EZH2 antibodies were applied at 1:100 dilution for 32 minutes at 36 °C, and POU2F3 antibodies were applied at 1:200 dilution for 64 minutes at 36 °C. IHC used a Ventana BenchMark Ultra platform (Roche, Tuscon, AZ, USA). Reactions were visualized using the OptiView Universal DAB Detection Kit (Roche) for EZH2 and the OptiView Amplification Kit (Roche) for POU2F3. Other parameters for IHC were described previously [9].

For EZH2 and POU2F3, the diffuseness of staining was independently scored by two pathologists (JRN, ACR) in 10% increments or as 0%, 1%, or 5% staining, across the whole tumor area. The single highest 200× tumor hotspot was also scored for POU2F3. For EZH2 only moderate or strong intensity nuclear staining was considered positive, whereas POU2F3 scoring considered nuclear staining of any intensity to be positive. In cases with scores that differed between the two pathologists by 30% or more, a consensus was achieved. For the remaining cases, the average of the two pathologist’s scores was used. CD5 and CD117 were scored by a single pathologist (ACR) as positive or negative using 50% and 10% thresholds, respectively, as described previously [9]. TdT-positive thymocytes were scored as present or absent, with rare scattered TdT-positive cells counted as absent [9]. The complete absence of nuclear staining for BAP1 and cytoplasmic and nuclear staining for MTAP immunostains was scored as loss, excluding cases lacking an internal positive control.

### 2.3. Statistical Analysis

Statistical analysis was performed using RStudio version 2022.12.0 build 353 and the R Project for Statistical Computing version 4.2.2 [19]. Interobserver agreement was assessed using Cohen’s kappa. Possible associations between positive staining with different markers were assessed using Fisher’s exact test. Wilcoxon rank sum tests with continuity correction were used to compare diffuseness scores between diagnosis groups, and to compare block age with positive versus negative staining. A *p*-value of <0.05 was considered significant. Receiver-operating characteristic (ROC) analysis was performed to aid in the determination of thresholds and to provide the “area under the curve” (AUC).

## 3. Results

### 3.1. Patients

This study included 37 thymic carcinomas (24 squamous cell carcinomas, 3 undifferentiated carcinomas, 2 small cell carcinomas, 2 adenocarcinomas, 2 mucoepidermoid carcinomas, 2 lymphoepithelial carcinomas, 1 sarcomatoid carcinoma, and 1 adenosquamous carcinoma) and 44 thymomas (23 type A thymomas, 13 type B3 thymomas, and 8 MNTLS). Patient demographics are summarized in Table 1. Older block age was not significantly associated with negative staining for any of the markers tested, or with thymic carcinoma diagnosis (Wilcoxon rank sum tests).

### 3.2. EZH2

**Figure 1:**
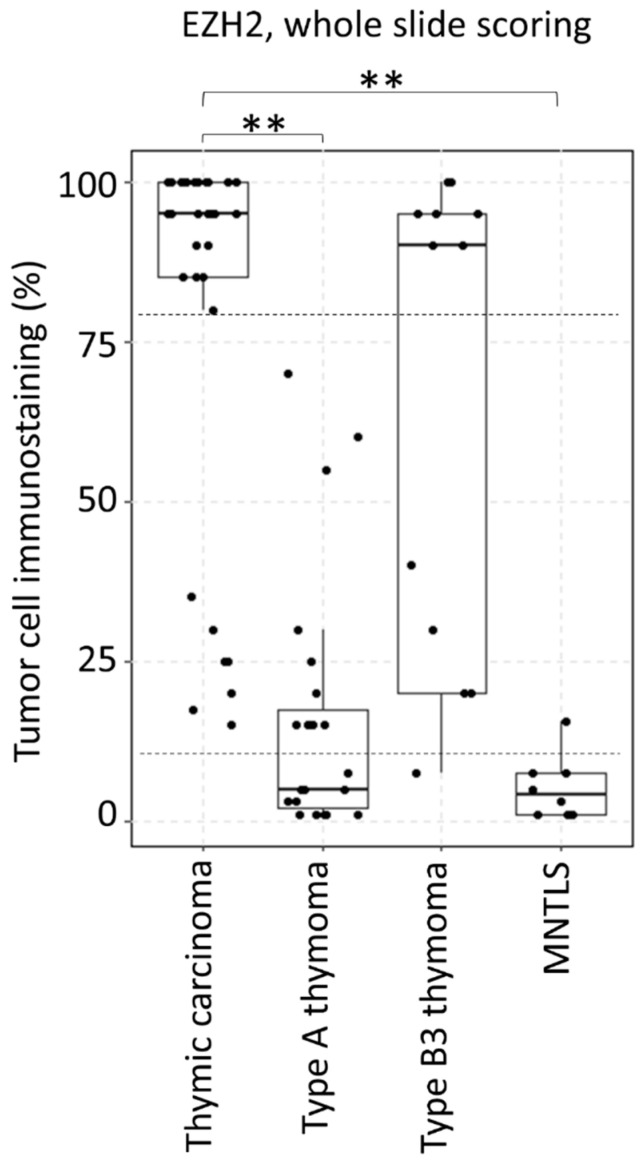
Diffuseness of EZH2 staining, scored across whole tissue slides. The dotted lines indicate 80% and 10% thresholds used for interpreting staining as positive or negative, respectively. ** indicates *p* < 0.001 in Wilcoxon rank sum tests. MNTLS, Micronodular thymoma with lymphoid stroma.

**Figure 2:**
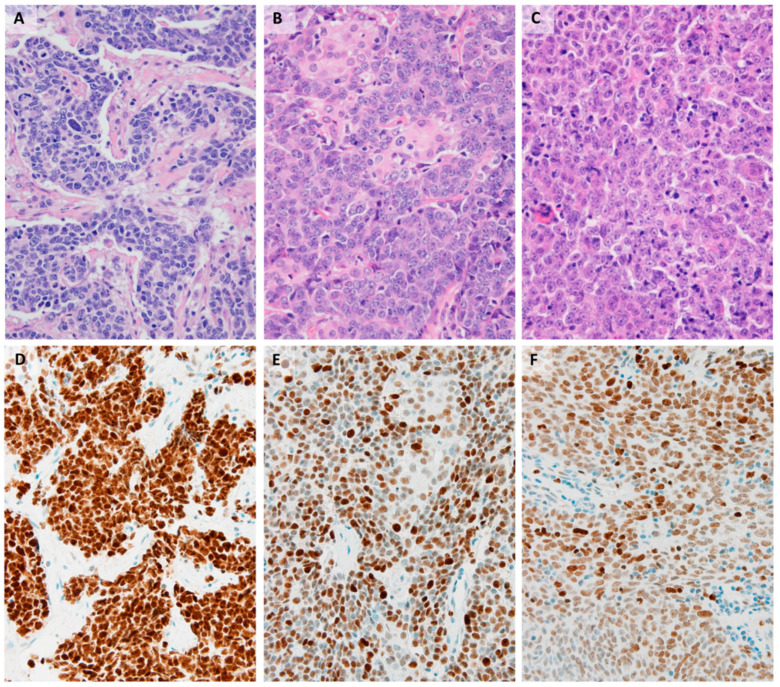
Hematoxylin and eosin (**A**–**C**) and EZH2 (**D**–**F**) immunostaining in thymic carcinomas ((**A**,**D**): small cell carcinoma; (**B**,**E**): squamous cell carcinoma) and a type B3 thymoma (**C**,**F**). All images are at 400× magnification and were scored as staining in ≥80% of tumor cells.

When considering the percent of tumor cells staining as a continuous variable, thymic carcinomas had significantly more diffuse EZH2 staining compared to type A thymoma (*p* < 0.0001) and MNTLS (*p* < 0.0001), but not when compared to type B3 thymoma (*p* = 0.055, Wilcoxon rank sum tests, Figure 1 and Figure 2). All (37/37) thymic carcinomas and 52% (23/44) of thymomas showed EZH2 staining in >10% of tumor cells. Fifty-four percent (7/13) of B3 thymomas but none of the type A thymomas or MNTLS showed EZH2 staining in ≥80% of tumor cells. Overall, EZH2 staining in ≥80% of tumor cells was 100% specific for thymic carcinoma versus type A thymoma and MNTLS, and 81% sensitive for thymic carcinoma of any subtype (Table 2). For the thymic squamous cell carcinoma subgroup, the sensitivity of EZH2 staining in ≥80% of tumor cells was 92%. The 7 (of 37) thymic carcinomas with EZH2 staining in <80% of tumor cells included 2 (of 24, 8%) squamous cell carcinomas, 2 (of 2, 100%) mucoepidermoid carcinomas, 2 (of 2, 100%) adenocarcinomas, and 1 (of 3, 33%) undifferentiated carcinomas.

ROC analysis of thymic carcinomas, type A thymomas, and MNTLS samples for optimal cut-point analysis (defined as the cut point that maximized the sum of the sensitivity and specificity) identified 80% as the optimal cut point, with sensitivity and specificity as described above. The AUC in this analysis was 0.97. Type B3 thymomas were excluded from ROC analysis as EZH2 staining overlapped considerably between carcinoma and B3 thymoma.

**Figure 3:**
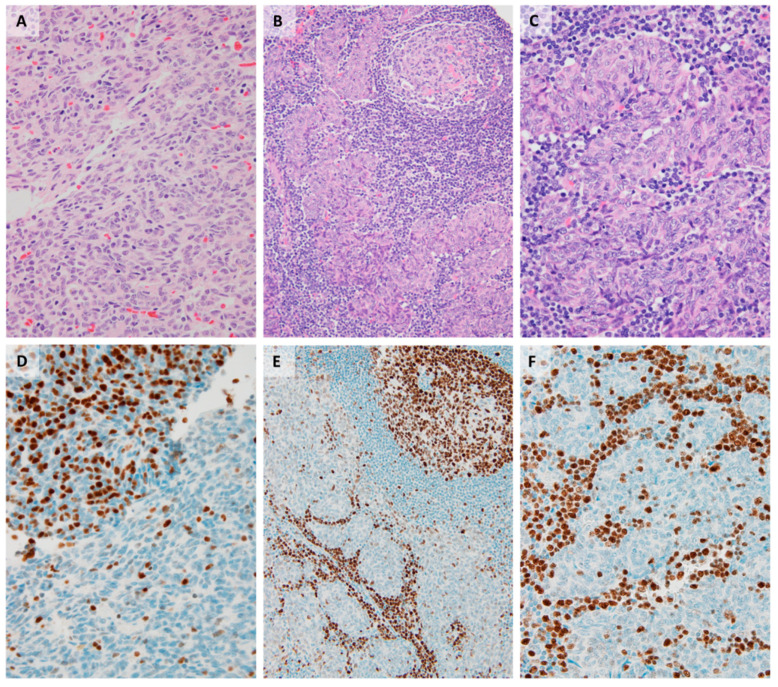
Hematoxylin and eosin (**A**–**C**) and EZH2 (**D**–**F**) immunostaining in a type A thymoma scored as <80% staining (**A**,**D**) and a micronodular thymoma with lymphoid stroma scored as <10% staining (**B**,**C**,**E**,**F**). Lymphocytes in (**E**,**F**) show patchy strong EZH2 staining. All images are at 400× magnification, except for (**B**,**E**) at 200× magnification.

Using the ≥80% threshold for EZH2 positivity, the two pathologists’ scores were concordant for all cases except for one squamous cell carcinoma and one type A thymoma (κ = 0.95, 95% CI 0.88–1, indicating excellent agreement). Considerable spatial heterogeneity in staining was noted, particularly in thymomas (Figure 3). Background lymphocytes including germinal center B-cells also showed patchy strong EZH2 staining (Figure 3).

### 3.3. POU2F3

**Figure 4:**
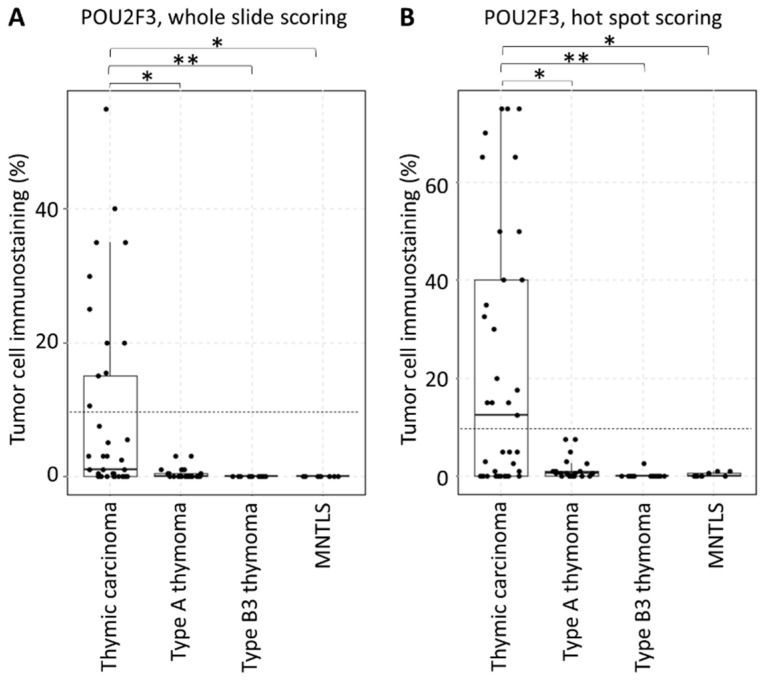
Diffuseness of POU2F3 immunostaining scored across the whole slide (**A**) and in a 200× hotspot (**B**). The dotted line indicates the 10% threshold used for interpreting staining as positive or negative. * indicates *p* < 0.01 and ** indicates *p* < 0.001 in Wilcoxon rank sum tests.

**Figure 5:**
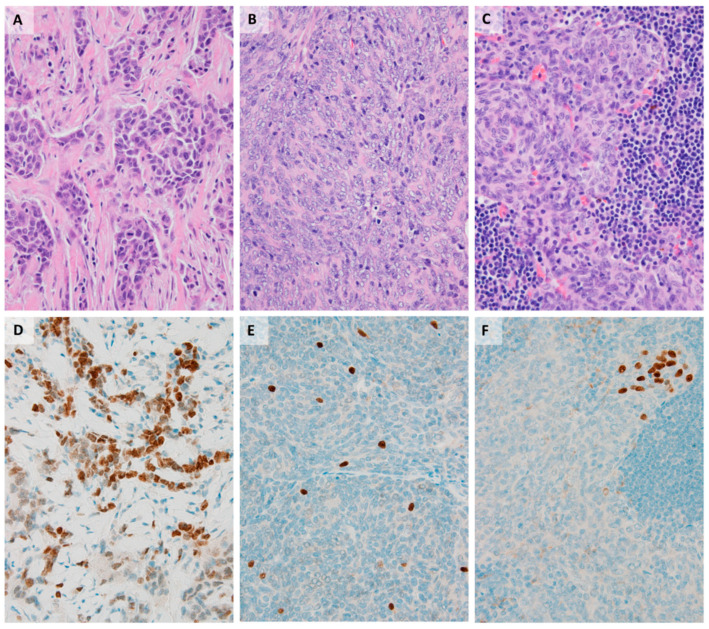
Hematoxylin and eosin (**A**–**C**) and POU2F3 (**D**–**F**) immunostaining in a thymic squamous cell carcinoma (**A**,**D**), a type A thymoma (**B**,**E**) and a micronodular thymoma with lymphoid stroma (**C**,**F**). The carcinoma shown in (**D**) was scored as staining in ≥10% of hotspot tumor cells, and the thymomas shown in (**E**,**F**) were scored as staining in <10% of hotspot tumor cells. All images are at 400× magnification.

When considering the percent of tumor cells staining as a continuous variable, POU2F3 staining was significantly more diffuse in thymic carcinomas than thymomas regardless of whether overall or hotspot scoring was used (*p* < 0.0001; Wilcoxon rank sum tests, Figure 4 and Figure 5). All thymomas showed staining in <10% of tumor cells using overall or hotspot scoring. Hotspot scores had 1–9% of tumor cell staining for 9 (of 23, 39%) type A thymomas, 2 (of 8, 25%) MNTLS, and 1 (of 13, 8%) type B3 thymoma, and were <1% in the remaining cases. Overall, POU2F3 staining in ≥10% of tumor cells was 100% specific for thymic carcinoma versus thymoma using overall or hotspot scoring. The sensitivity of ≥10% POU2F3 staining for thymic carcinoma was 30% using overall scoring, and 51% using hotspot scoring (Table 2). Sensitivity for the thymic squamous cell carcinoma subgroup was 46% using overall scoring and 58% using hotspot scoring (Table 2). There was disagreement between the two pathologists across the 10% threshold for hotspot scoring of 7 (of 37, 19%) carcinomas and 2 (of 44, 5%) thymomas (κ = 0.68, 95% CI 0.49–0.87, indicating substantial agreement). All but two of the carcinomas with ≥10% POU2F3 hotspot staining also had EZH2 staining in ≥80% of tumor cells.

ROC analysis had an AUC of 0.77 and identified 3% as the cut point that maximized the sum of the sensitivity and specificity (i.e., 62% sensitivity and 91% specificity using the 3% threshold). Compared to the 10% threshold described above, a 3% threshold decreased the specificity while producing a small gain in sensitivity. To maximize specificity, the 10% threshold was used in the analysis below.

### 3.4. Comparison of EZH2, POU2F3, CD5, CD117, TdT, BAP1, and MTAP Immunoreactivity

Results for CD117, CD5, BAP1, MTAP, and TdT are similar to those previously published [9], as 32 out of 37 carcinomas and all thymomas in the present study were included in the prior study. The specificity of CD117 and CD5 for thymic carcinoma versus thymoma was 100%, and the sensitivity of these markers for thymic carcinoma was 86% and 35%, respectively (Table 3). Two of the five carcinomas negative for CD117 were positive for EZH2 using the ≥80% threshold (both squamous cell carcinomas), such that performing both EZH2 and CD117 IHC increased sensitivity from 86% (for CD117 alone) to 92%. All carcinomas positive for POU2F3 were also positive for CD117, such that performing IHC for both POU2F3 and CD117 did not increase sensitivity above that of CD117 alone. Positive POU2F3 and CD117 staining significantly co-occurred (*p* = 0.02, Fisher’s exact test), as did positive POU2F3 and CD5 staining (*p* = 0.005, Fisher’s exact test).

Loss of BAP1 and MTAP1 immunoreactivity was 100% specific for thymic carcinoma versus thymoma and occurred in 11% (4/36) and 14% (5/35) of the evaluable carcinomas, respectively (Table 3). Two of the five cases with MTAP loss showed a geographic area of negative staining, consistent with subclonal loss. The carcinomas with BAP1 or MTAP loss included one adenocarcinoma with <80% EZH2 staining and negative CD117 staining, such that the addition of BAP1 and MTAP to a panel of EZH2 (≥80% threshold) and CD117 slightly increased the sensitivity for carcinoma from 92% to 95%. However, if EZH2 (≥80%) is the only positive stain, B3 thymoma would not be excluded, given the moderate proportion of B3 thymomas (54%) that had EZH2 staining in ≥80% of tumor cells. Interestingly, loss of BAP1 was significantly associated with <80% EZH2 staining (*p* = 0.018, Fisher’s exact test).

Clusters of TdT-positive thymocytes, suggestive of thymoma rather than thymic carcinoma, had a sensitivity of 77% (34/44 thymomas positive) and a specificity of 97% for thymoma versus thymic carcinoma (1/37 carcinomas positive) [9]. Of the 10 thymomas (including 6 type A and 4 type B3 thymomas) that lacked TdT-positive thymocytes, 5 had EZH2 staining in <10% of tumor cells, which would help to exclude the possibility of carcinoma.

We then explored how adding TdT to a panel of EZH2, CD117, BAP1, and MTAP affected the proportion of TETs for which a potentially informative result was obtained. We defined a potentially informative result as one that had ≥97% specificity in our data and summarized the results in Table 4. As CD5 and POU2F3 stained only CD117 positive carcinomas, and therefore would not improve sensitivity over that of CD117, they are not included in our recommended panel. Across all TETs in our study, informative results were obtained for 95% (77/81) of cases using a panel of TdT, EZH2, CD117, BAP1 and MTAP. Without BAP1 and MTAP, informative results would still have been obtained for 94% (76/81) of cases, whereas without EZH2 informative results would have been obtained for only 83% (67/81) of cases. In only one case conflicting informative results were obtained, a small cell carcinoma with both CD117 and TdT positivity. Given the superior specificity of CD117, we would favor interpretation of such a result as supporting carcinoma.

## 4. Discussion

Our study provides an independent assessment of two immunostains with potential utility for the distinction of thymic carcinoma from thymoma. EZH2 staining using a ≥80% positivity threshold was 100% specific for thymic carcinoma versus type A thymoma and MNTLS but was not useful for the distinction of thymic carcinoma from type B3 thymoma. EZH2 used alone had 81% sensitivity for thymic carcinoma (using the ≥80% threshold), which was higher than the sensitivity of CD5 (35%) but lower than the sensitivity of CD117 (86%). The addition of EZH2 increased the overall sensitivity for thymic carcinoma to 92% when used in combination with CD117. As all thymic carcinomas showed EZH2 staining in >10% of tumor cells, staining in ≤10% of tumor cells may provide evidence against the possibility of thymic carcinoma. POU2F3 hotspot staining using a ≥10% positivity threshold had 100% specificity for thymic carcinoma versus thymoma but had a sensitivity of only 51%. All cases positive for POU2F3 were also positive for CD117, such that adding POU2F3 to a panel containing CD117 would not improve sensitivity.

To our knowledge, our study is the first to assess EZH2 in non-squamous and non-neuroendocrine thymic carcinomas. We provide the first report of ≥80% EZH2 staining in thymic lymphoepithelial, sarcomatoid, undifferentiated, and adenosquamous thymic carcinomas. The only prior study of EZH2 for thymic carcinoma versus thymoma used tissue microarrays to assess EZH2 in thymic squamous cell carcinomas (*n* = 27) and B3 thymomas (*n* = 26) [4]. In their dataset, EZH2 staining in ≥10% of tumor cells had 89% sensitivity and 100% specificity for thymic squamous cell carcinoma versus B3 thymoma. This contrasts with the poor specificity of EZH2 for carcinoma versus B3 thymoma in our study, and may relate to sampling bias (given the use of tissue microarrays in the prior study) and the use of different antibodies (clone 6A10 from Novocastra used by Kim et al. [4] versus clone D2C9 from Cell Signaling Technology, used in our study). Although EZH2 is mentioned in the current WHO as useful for distinguishing B3 thymoma and thymic carcinoma [17], to our knowledge, this conclusion was only supported by the one prior study [4]. This discrepancy between the prior study and our study regarding the utility of EZH2 for distinguishing type B3 thymoma from thymic carcinoma highlights the value of independent validation of emerging markers prior to their routine clinical use, and the value of providing antibody clones in recommendations. EZH2 immunoreactivity has also been reported in thymic neuroendocrine neoplasms, with one study showing >25% EZH2 staining in 83% (5/6) of thymic large cell neuroendocrine carcinomas and 0/5 carcinoid tumors [20], and a separate study reporting >1% EZH2 staining in 37% (10/27) of thymic carcinoid tumors [21]. These data are in keeping with ≥80% EZH2 staining in both small cell carcinomas in our study.

Applications of EZH2 IHC have also been explored for mesothelial [22,23], breast [24], prostate [25], biliary [26], endocervical [27], and liver [28] neoplasms, and therefore EZH2 may become a multi-purpose addition to a laboratory’s available immunostains. EZH2 staining of lymphocytes may be useful as an internal positive control but must be carefully distinguished from tumor cell staining. Lymphocyte rich tumors such as B1 and B2 thymomas were not included in our study as they are in general not in the differential diagnosis of thymic carcinoma. We also caution that EZH2 staining in thymomas showed considerable heterogeneity. Coincidental sampling of only strongly staining areas in a small biopsy may result in a false impression of overall EZH2 positivity.

The variability in EZH2 staining in TETs has implications for the development of targeted therapies. The EZH2 inhibitor tazemetostat has not yet been trialed in TETs [29] but is being explored for other malignancies, with promising results from early phase trials [30]. Because EZH2 is a histone modifying enzyme, EZH2 levels could potentially impact the response to agents targeting other histone modifiers, such as the pan-histone deacetylase inhibitor belinostat [4]. Combination belinostat and chemotherapy treatment produced objective responses in 64% of thymomas and 21% of thymic carcinomas in a phase I/II trial [31]. Molecular features were not used for patient selection, such that heterogeneity in levels of EZH2 and other epigenetic regulators may have contributed to variability in treatment response. Recurrent mutations in chromatin remodeling genes in thymic carcinoma implicate epigenetic dysregulation in the pathogenesis of thymic carcinoma [32,33], but the precise mechanism through which aberrant EZH2 expression may contribute to the development of TETs remains to be investigated. Interestingly, BAP1 also plays a role in the regulation of histone modifications, and therefore BAP1 status may also impact response to EZH2 inhibitors and histone deacetylase inhibitors [34,35]. In mesothelioma, BAP1 loss promotes EZH2 expression [34], but the opposite association was seen in our data (i.e., BAP1 loss was significantly associated with <80% EZH2 expression).

We found a substantial but not excellent interobserver agreement (κ = 0.68) in the interpretation of POU2F3 staining, likely due to the considerable proportion of cases with staining near the 10% threshold for positivity, differences in the areas selected for hot spot scoring, and differences in whether very weak staining was considered real or artefactual. The degree of interobserver agreement for POU2F3 is within the range seen for commonly used clinical immunostains (e.g., κ = 0.63 for PD-L1 around the 1% threshold [36], κ = ~0.6 for Ki-67 with cut-offs in the 10–20% range [37] and κ = 0.65 for HER2 0/1+ vs. 2/3+ [38]). Caution is required in the interpretation of results near the cut-off value, where interobserver variability may be the greatest.

The frequency of POU2F3 immunoreactivity in our study was similar to that reported in two prior studies. One study using tissue microarrays found moderate to strong POU2F3 staining in >40% of tumor cells in 72% (18/25) of thymic squamous cell carcinomas, 8% (1/12) of type B3 thymomas, and none (0/10) of the type A thymomas [16]. A subsequent study (using both tissue microarrays and whole tissue sections) reported POU2F3 staining of >10% of hotspot tumor cells in 56% (9/16) of thymic squamous cell carcinomas, 8% (1/12) of type A thymomas, and none (0/22) of the MNTLS and B3 thymomas [15], which was similar to our findings.

As proposed previously, the presence of POU2F3 immunoreactivity may indicate the acquisition of a tuft cell phenotype by thymic carcinomas [16]. As tuft cells exist in the thymic medulla [13,14], the presence of rare POU2F3 positive cells in type A thymomas and MNTLS may be consistent with a medullary origin of these tumors [15]. CD117 has also been implicated in the determination of tuft cell identity in the thymus, and an association between POU2F3 and CD117 has previously been reported [16], consistent with the co-occurrence of positive staining for these markers in our study. L1CAM, another marker of tuft cell differentiation, also showed excellent specificity and moderate sensitivity for thymic carcinoma in the prior study [15].

We also detected POU2F3 immunoreactivity in mucoepidermoid (1/2) and lymphoepithelial (2/2) carcinomas of the thymus, which to our knowledge is the first immunohistochemical detection of POU2F3 in these carcinoma subtypes. One case of thymic mucoepidermoid carcinoma previously tested for POU2F3 was reported to be negative [15]. Our findings add these tumor types to a growing list of neoplasms that can show POU2F3 immunoreactivity, including pulmonary small cell carcinoma, large cell neuroendocrine carcinoma, and basaloid squamous cell carcinoma [12]. An association between POU2F3 mRNA expression and sensitivity to the chemotherapeutic agent lurbinectedin was demonstrated using pulmonary small cell carcinoma cell lines [39], raising the possibility that POU2F3 may be predictive of treatment response. This possibility remains to be investigated for TETs.

Limitations of our study include the length of time that some blocks were in storage, as antigenicity may decrease over time. Although using whole sections allowed assessment of staining heterogeneity and reduced sampling error, the heterogeneity of staining in large tissue sections may be affected by uneven fixation. Furthermore, the small number of cases available for rare thymic carcinoma subtypes limits our understanding of the sensitivity of immunohistochemical markers for these tumors. We also caution that EZH2 and POU2F3 are detectable in non-thymic carcinomas [12,22,23,24,25,26,27,28], and immunoreactivity for these markers does not imply thymic origin.

## 5. Conclusions

EZH2 may be a valuable addition to immunohistochemical panels to distinguish thymic carcinomas from thymoma. EZH2 staining in ≤10% of tumor cells may help to exclude the possibility of thymic carcinoma. EZH2 staining in ≥80% of tumor cells may provide evidence against type A thymoma and MNTLS but does not reliably distinguish thymic carcinoma from type B3 thymoma. POU2F3, although highly specific for carcinoma versus thymoma, has moderate sensitivity and shows strong overlap with CD117 staining. Overall, our findings underscore heterogeneity in key regulators of gene expression in TETs, with implications for our understanding of tumor histogenesis and the development of targeted therapies.

## Figures and Tables

**Table 1:** Patient demographics and WHO classification of thymic epithelial tumors.

Diagnosis	Total Number of Cases	Resections *n* (%)	Biopsies *n* (%)	Male *n* (%)	Patient Age at Time of Specimen Collection, Years, Median (Range)	Tissue Block Age at Time of Immunostaining, Years, Median (Range)
**Carcinoma overall**	**37**	**36 (97)**	**1 (3)**	**21 (57)**	**53 (19–79)**	**11 (0.2–59)**
Squamous cell carcinoma	24	23 (96)	1 (4)	12 (50)	57.5 (48–79)	10 (2–27)
Undifferentiated carcinoma	3	3 (100)	0 (0)	2 (67)	72 (38–75)	1 (0.2–13)
Small cell carcinoma	2	2 (100)	0 (0)	0 (0)	48.5 (41–56)	2.5 (2–3)
Adenocarcinoma	2	2 (100)	0 (0)	2 (100)	56.5 (42–71)	18.5 (15–22)
Mucoepidermoid carcinoma	2	2 (100)	0 (0)	2 (100)	54 (39–69)	9.5 (3–16)
Lymphoepithelial carcinoma	2	2 (100)	0 (0)	1 (50)	25 (19–31)	19 (11–27)
Sarcomatoid carcinoma	1	1 (100)	0 (0)	1 (100)	45	2
Adenosquamous carcinoma	1	1 (100)	0 (0)	1 (100)	52	15
**Thymoma overall**	**44**	**44 (100)**	**0 (0)**	**16 (36)**	**63.5 (28–87)**	**8 (0.4–25)**
Type A	23	23 (100)	0 (0)	10 (43)	64 (38–83)	11 (1–20)
Type B3	13	13 (100)	0 (0)	4 (31)	53 (28–85)	6 (2–25)
Micronodular thymoma with lymphoid stroma	8	8 (100)	0 (0)	2 (25)	77 (61–87)	7 (0.4–11)

**Table 2:** Proportion of cases with positive staining for EZH2 and POU2F3.

Diagnosis	Total Number of Cases	EZH2		POU2F3	
		Overall, ≥80% Threshold *n* (%)	Overall, >10% Threshold *n* (%)	Overall, ≥10% Threshold *n* (%)	Hotspot, ≥10% Threshold *n* (%)
**Carcinoma overall**	**37**	**30 (81)**	**37 (100)**	**11 (30)**	**19 (51)**
Squamous cell carcinoma	24	22 (92)	24 (100)	11 (46)	14 (58)
Undifferentiated carcinoma	3	2 (67)	3 (100)	0 (0)	2 (67)
Small cell carcinoma	2	2 (100)	2 (100)	0 (0)	0 (0)
Adenocarcinoma	2	0 (0)	2 (100)	0 (0)	0 (0)
Mucoepidermoid carcinoma	2	0 (0)	2 (100)	0 (0)	1 (50)
Lymphoepithelial carcinoma	2	2 (100)	2 (100)	0 (0)	2 (100)
Sarcomatoid carcinoma	1	1 (100)	1 (100)	0 (0)	0 (0)
Adenosquamous carcinoma	1	1 (100)	1 (100)	0 (0)	0 (0)
**Thymoma overall**	**44**	**10 (23)**	**23 (52)**	**0 (0)**	**0 (0)**
Type A	23	0 (0)	10 (43)	0 (0)	0 (0)
Type B3	13	7 (54)	12 (92)	0 (0)	0 (0)
Micronodular thymoma with lymphoid stroma	8	0 (0)	1 (13)	0 (0)	0 (0)

**Table 3:** Proportion of cases with positive staining for single or combined immunostains.

	Thymic Carcinomas		Thymomas		
Immunostain	Of Any Histologic Type (*n* = 37) *n* (%)	Squamous Cell Carcinoma (*n* = 24) *n* (%)	Type A (*n* = 23) *n* (%)	Type B3 (*n* = 13) *n* (%)	MNTLS (*n* = 8) *n* (%)
POU2F3 ^1^	19 (51)	14 (58)	0 (0)	0 (0)	0 (0)
EZH2 ^2^	30 (81)	22 (92)	0 (0)	7 (54)	0 (0)
CD117	32 (86)	22 (92)	0 (0)	0 (0)	0 (0)
CD5	13 (35)	11 (46)	0 (0)	0 (0)	0 (0)
BAP1 loss	4 (11)	1 (4)	0 (0)	0 (0)	0 (0)
MTAP loss	5 (14)	2 (8)	0 (0)	0 (0)	0 (0)
TdT-positive thymocytes	1 (3)	0 (0)	17 (74)	9 (69)	8 (100)
CD117 or CD5	32 (86)	22 (92)	0 (0)	0 (0)	0 (0)
CD117 or POU2F3 ^1^	32 (86)	22 (92)	0 (0)	0 (0)	0 (0)
CD117 or EZH2 ^2^	34 (92)	24 (100)	0 (0)	7 (54)	0 (0)
CD117, MTAP loss, or BAP1 loss	33 (89)	22 (92)	0 (0)	0 (0)	0 (0)
CD117, EZH2 ^2^, MTAP loss, or BAP1 loss	35 (95)	24 (100)	0 (0)	7 (54)	0 (0)

MNTLS, Micronodular thymoma with lymphoid stroma. ^1^ POU2F3 scoring used a ≥10% threshold and hotspot scoring ^2^ EZH2 used a ≥80% threshold.

**Table 4:** Suggested interpretation of potentially informative results from the recommended panel.

Marker	Result	Suggested Interpretation ^1^	Evidence
EZH2	≤10% tumor cell staining	Thymoma	100% specificity for thymoma versus thymic carcinoma
EZH2	≥80% tumor cell staining	Thymic carcinoma or Type B3 thymoma	100% specificity for thymic carcinoma or Type B3 thymoma versus Type A thymoma or MNTLS
CD117	≥10% tumor cell staining	Thymic carcinoma	100% specificity for thymic carcinoma versus thymoma
BAP1	Complete loss of nuclear staining in tumor cells	Thymic carcinoma	100% specificity for thymic carcinoma versus thymoma
MTAP	Complete loss of cytoplasmic and nuclear staining in tumor cells	Thymic carcinoma	100% specificity for thymic carcinoma versus thymoma
TdT	More than rare scattered thymocytes with positive staining	Probable thymoma	97% specificity for thymoma versus thymic carcinoma

^1^ Assuming a morphologic differential diagnosis of thymic carcinoma versus type A thymoma, B3 thymoma or micronodular thymoma with lymphoid stroma, and excluding entities other than TETs.

## Data Availability

The data presented in this study is available in this article.
